# The Decline of Mechanical Dentistry—Its Appropriateness

**Published:** 1877-11

**Authors:** 


### EDITORIAL, ETC.

*The Decline of Mechanical Dentistry.—Its Appropriateness.*—We publish elsewhere an abstract from an article by Prof. Barker, on the above subject. The points he makes are forcibly put, and seem at least partly true. Here however as elsewhere the doctors radically differ. People will continue to lose their teeth, possibly faster in the future than in the past, owing to causes entirely beyond the control of the dentists. Until we are so regenerated physically as to have teeth of better construction, more perfectly calcified, and until our patients can be educated to take better care of the work we do for them,—and even with the best they can do the inherent difficulties of perfectly cleansing teeth are almost insurmountable,—until much that now seems inevitable can be overcome, we may not hope or expect but that artificial dentistry must hold a prominent place in the profession. It is useless to sigh for the day when all natural teeth will be preserved. So long as, to use the words of a writer, "children are begotten in debility and born in feebleness, so long as nervous and overworked fathers, and ænemic and immature mothers, bring illy nourished and scrofulous children into the world, to perish prematurely or drag out a life of feebleness and enervation," so long may we expect to furnish such patients with artificial substitutes for their perishing natural organs.

We have not felt however, like joining in the cry as to the appropriateness of the decline of mechanical dentistry. That it has declined, is true enough, but this degradation is due not so much to anything inherent in this art of substitution, as to its importance being overlooked in the anxiety to perform difficult and doubtful operations. We have no disposition to underrate the beautiful fillings which are seen very often now, but it must be apparent to every reflective mind that often these operations, splendid works of art intrinsically considered, are as much out of harmony with nature as is possible. We are satisfied that the day will come when the glitter of contour fillings will be looked upon as a relic of the days when the æsthetic in dentistry was in its crudity, raw and unrefined. When this is realized, and when the profession is sufficiently advanced to feel that the glare and glitter of a flashy gold tooth is not in good taste, the inappropriateness of the "decline" of dental mechanism will be better seen and understood.

We should feel mortified at the failure of our operations only, it seems to us, as we realize that these failures are due to our imperfect work. We should not lose sight of the fact that many teeth are, from causes above mentioned, utterly unsalvable, and that their total loss is only a question of time; and while making no excuses for imperfect work we know that many of the "best men" sigh over the wreck of their best efforts at conservation.

In no department of surgery does the operator so confidently promise his patient good and lasting results as in dentistry, and it is to over-confidence in the durability of what we do that the mischief lies. If dentists would do less of this assurance of immunity from decay, it would be wiser; for reflection and observation should make us sadly conscious that we cannot do so consistently, and the failure in such case is made by the patient to be the fault of the operator. There is no disposition here to underrate the inestimable value of good fillings, only to press the truth that our work is reparative only, and as snch should not be promised for too certainly. The probability is almost a certainty that in the future the necessity for artificial teeth will increase instead of diminish, and that the few earnest men in that department will have accessions to their ranks instead of desertions. It remains for the profession to correctly teach the public who require teeth of replacement what true art is, and endeavor to overcome the utter perversion of taste, which allows and even calls for the present barbarous glitter of artificial teeth truly called "false." H.

